# Use of podcasts for health education: a scoping review

**DOI:** 10.1590/0034-7167-2023-0096

**Published:** 2024-03-15

**Authors:** Fabiola Leticia Damascena Amador, Gabriele Cardoso Gonçalves Alves, Vagner Rogério dos Santos, Rita Simone Lopes Moreira

**Affiliations:** I Universidade Federal de São Paulo. São Paulo, São Paulo, Brazil

**Keywords:** Health Education, Patient Education as Topic, Webcasts, Telemedicine, Audiovisual Aids, Educación en Salud, Educación del Paciente como Asunto, Difusión por la *Web*, Telemedicina, Recursos Audiovisuales, Educação em Saúde, Educação de Pacientes como Assunto, Webcast, Telemedicina, Recursos Audiovisuais

## Abstract

**Objectives:**

to map the scientific evidence related to the characteristics, themes, and outcomes of using health education podcasts aimed at individuals over 18 years of age in intra or extrahospital environments.

**Methods:**

a scoping review, based on the Joanna Briggs Institute method, conducted in 11 databases, including studies from 2004 to 2022.

**Results:**

11 studies were selected, categorized, highlighting the characteristics, evaluated outcomes, areas, and conditions of podcast application, indicating it as an effective tool for promoting behavioral change, health promotion, and social interaction, demonstrating its potential to improve well-being, quality of life, and user/client autonomy.

**Conclusions:**

the use of podcasts proves to be an effective, innovative, and low-cost tool, with a significant social impact, being effective for behavioral change, satisfaction, and social interaction. However, the lack of comprehensive studies on podcast development methodologies represents challenges to be overcome.

## RESUMO

**Objetivos:**

mapear as evidências científicas relacionadas às características, temáticas e desfechos do uso de *podcasts* de educação em saúde direcionados para indivíduos maiores de 18 anos nos ambientes intra ou extrahospitalares.

**Métodos:**

revisão de escopo, baseando-se no método do *Joanna Briggs Institute*, realizada em 11 bases de dados, incluindo estudos de 2004 a 2022.

**Resultados:**

foram selecionados 11 estudos, categorizados e destacando as características, desfechos avaliados, áreas e condições de aplicação do *podcast*, apontando-o como uma ferramenta eficaz para promover a mudança comportamental, a promoção da saúde e a interação social, evidenciando seu potencial para melhorar o bem-estar, qualidade de vida e autonomia dos usuários/clientes.

**Conclusões:**

o uso do *podcast* demonstra ser uma ferramenta eficaz, inovadora e de baixo custo, com impacto social significativo, sendo eficaz para mudança comportamental, satisfação e interação social. No entanto, a falta de estudos abrangentes sobre as metodologias de desenvolvimento de *podcasts* representam desafios a serem superados.

## RESUMEN

**Objetivos:**

mapear las evidencias científicas relacionadas con las características, temáticas y resultados del uso de *podcasts* de educación en salud dirigidos a individuos mayores de 18 años en entornos intra o extrahospitalarios.

**Métodos:**

revisión de alcance, basada en el método del *Joanna Briggs Institute*, realizada en 11 bases de datos, incluyendo estudios de 2004 a 2022.

**Resultados:**

se seleccionaron 11 estudios, categorizados y destacando las características, resultados evaluados, áreas y condiciones de aplicación del *podcast*, señalándolo como una herramienta eficaz para promover el cambio de comportamiento, la promoción de la salud y la interacción social, evidenciando su potencial para mejorar el bienestar, la calidad de vida y la autonomía de los usuarios/clientes.

**Conclusiones:**

el uso del *podcast* demuestra ser una herramienta eficaz, innovadora y de bajo costo, con un impacto social significativo, siendo eficaz para el cambio de comportamiento, la satisfacción y la interacción social. Sin embargo, la falta de estudios exhaustivos sobre las metodologías de desarrollo de *podcasts* representa desafíos a superar.

## INTRODUCTION

Health education emerges as a collaborative process that encompasses the guidance of health professionals to individuals, with the aim of fostering the development of skills and behaviors focused on optimizing health outcomes^(1)^. Through this approach, patient education can have a direct influence on their behavioral patterns, inciting changes in the domains of knowledge, attitude, and essential skills for improving their health status^(2)^.

In this context, the active participation of individuals in the educational process, following pedagogical and andragogical approaches, emerges as a valuable tool in managing health conditions. However, the actions implemented and processes proposed so far have not demonstrated the necessary efficacy^(3)^.

Thus, the implementation of strategies aimed at patient engagement, capable of promoting a deep understanding of the health-disease relationship and enabling the precise application of acquired knowledge, emerges as a highly promising approach in this educational process^(4)^. It is also essential to rethink conventional educational approaches, as traditional methods have shown limitations in meeting contemporary demands^(5)^.

Digital forms, such as videos or podcasts, have increasingly been explored as narrative communication and health education resources for adults and the elderly^(6)^. This is because, by immersing individuals in stories, such resources assist in information processing and overcoming behavioral obstacles^(7)^. In this context, narratives can evoke more intense emotional responses compared to the mere presentation of information^(8)^, increase the patient’s listening capacity^(9)^, and trigger the self-reference effect, facilitating the recall of facts in which the individual was involved^(10)^. Thus, according to Fisher’s theory^(11)^, research has shown that conveying information through narratives engages participants more deeply, promotes positive attitudes, increases knowledge, and strengthens the behavioral intention to adopt healthy changes^(7,12-14)^.

In this scenario, podcast technology emerges as highly relevant-a digital communication medium that allows for the recording of narrations or interviews, akin to radio programs^(15)^. It presents itself as a versatile and dynamic resource for sharing health information^(16)^. However, despite the abundance of health-related podcasts available for download, their usage remains largely unexplored^(17)^.

Therefore, podcasts have the potential to become a promising resource in enhancing the effectiveness of health education strategies. However, they remain underutilized and underexplored in the literature. In this context, a scoping review was proposed to explore the available literature and map scientific evidence relating to the use of podcasts in the health education of adults and the elderly.

## OBJECTIVES

To map the scientific evidence related to the characteristics, themes, and outcomes of using health education podcasts aimed at individuals over 18 years of age in intra or extrahospital environments.

## METHODS

### Ethical Considerations

As this study is a review, it did not require approval from an Ethics Research Committee.

### Study Design

This scoping review was conducted following the JBI methodology for scoping reviews^(18)^ and adhered to the Preferred Reporting Items for Systematic Reviews and Meta-Analyses for Scoping Reviews (PRISMA-ScR) reporting standards^(19)^. It originated from a master’s dissertation and was conducted in the following stages from July to September 2022: preliminary literature review; development of the title, research question, and objectives; determination of eligibility criteria; review protocol development; search strategy development; study selection; data extraction; critical analysis of studies; presentation of results; and discussion of findings.

### Methodological Procedure

For the development of this study, a preliminary search was conducted in the MEDLINE, Cochrane Database of Systematic Reviews, and JBI Evidence Synthesis databases, which did not identify any systematic or scoping reviews, conducted or ongoing, related to the use of podcasts as a patient education strategy. Subsequently, the protocol for this scoping review was drafted and published on the Open Science Framework (OSF) in August 2022 with the identifier DOI 10.17605/OSF.IO/DQ4J7^(20)^. Minor changes were made to the population, objective, research question, and source of evidence for this review, in accordance with the JBI methodology. Due to the iterative nature of scoping reviews, protocol changes may be necessary^(18)^.

The title, objectives, and review question were formulated following the Population, Concept, and Context (PCC) mnemonic. Therefore, the guiding question of the study was: What are the characteristics, themes, and outcomes related to the use of health education podcasts targeted at individuals over 18 years old in intra or extrahospital settings?

### Inclusion Criteria

#### Types of Participants

As the target population, we chose to include evidence from studies involving individuals over 18 years old^(20)^.

#### Concept

Studies that considered the development and/or use of podcasts for health education, taking into account the characteristics of the tool, the health themes addressed by the podcast, and the outcomes evaluated after the intervention, were included^(20)^.

#### Context

Studies addressing health education in intra or extrahospital settings, without gender, ethnicity, or location distinctions, were included^(20)^.

#### Types of Evidence Sources

We considered studies available for free, as well as paid access, only in online format, such as systematic reviews, integrative reviews, scoping reviews, state-of-the-art reviews, primary research studies, meta-analyses, guidelines, experimental and epidemiological studies, including randomized and non-randomized controlled clinical trials, quasi-experimental studies, before-and-after studies, prospective and retrospective cohorts, case-control studies, cross-sectional studies, opinion articles, qualitative studies, reviews, theses, and dissertations^(20)^. We included studies published in English, Portuguese, and Spanish from 2004 to 2022. Conference abstracts, study protocols, editorials, comments, reviews, letters, websites, blogs, and studies not available in full were excluded^(20)^.

#### Search Strategy

The search strategy aimed to locate studies published as well as gray literature and was conducted in August 2022.

Initially, a preliminary limited search was performed with the keywords “patient education” AND “podcast” in the Pubmed and BVS Portal databases to identify potential studies by two reviewers independently. After this selection, a new reading of titles, abstracts, and keywords was carried out, considering the indexed terms and DeCS/MeSH descriptors for developing the search strategy adapted to each database^(19)^: (“Webcasts as Topics” OR “Podcasts as Topic” OR “Podcasts as Topics” OR “media”) AND (“health information” OR “knowledge” OR “learning” OR “communication”) AND (“Education, Patient” OR “Patient Education” OR “Education of Patients” OR “Education, Health” OR “Community Health Education” OR “Education, Community Health” OR “Health Education, Community”). The databases used included Cochrane Library, BVS Portal, PubMed, CINAHL, Web of Science, the Open Grey System of Grey Literature in Europe, Brazilian Library of Theses and Dissertations, Catalog of Theses and Dissertations, Open Access Theses and Dissertations, and Networked Digital Library of Theses and Dissertations.

We considered studies published in English, Portuguese, and Spanish, published between 2004 and 2022 because the term “podcast” only emerged after 2004, becoming a decentralized and free medium for independent communication^(15,21)^.

#### Study Selection

After searching all databases, all identified studies were uploaded to the Rayyan platform^(22)^ for screening, full-text reading, and study selection by two independent reviewers. After selecting the studies, the reference lists of all included studies were analyzed to search for additional studies. Any disagreements between the reviewers were resolved through discussion and consensus between them and a third, more experienced reviewer^(20)^.

#### Data Extraction

Data extraction from the included studies was performed by one reviewer using the standardized JBI instrument^(18)^ revised and modified by the reviewers according to the study question, from August to September 2022. The other reviewer subsequently analyzed the data for verification.

#### Critical Analysis of Studies

To assess the methodological quality of the studies, including trustworthiness, relevance, and outcomes, the JBI Critical Appraisal Checklist^(23)^ was used to support the analysis of the study’s design and conduct and assist in the interpretation of results, as it allows for the calculation of the risk of bias percentage^(24-25)^. According to JBI methodology, this analysis is not mandatory but enhances the reliability of the results^(18)^.

For this review, two reviewers used four checklists that cover the types of studies included: a checklist for analytical cross-sectional studies, a checklist for quasi-experimental studies, the JBI Critical Appraisal Tool for Randomized Controlled Trials, and a checklist for text and opinion^(23)^.

## RESULTS

**Figure 1:**
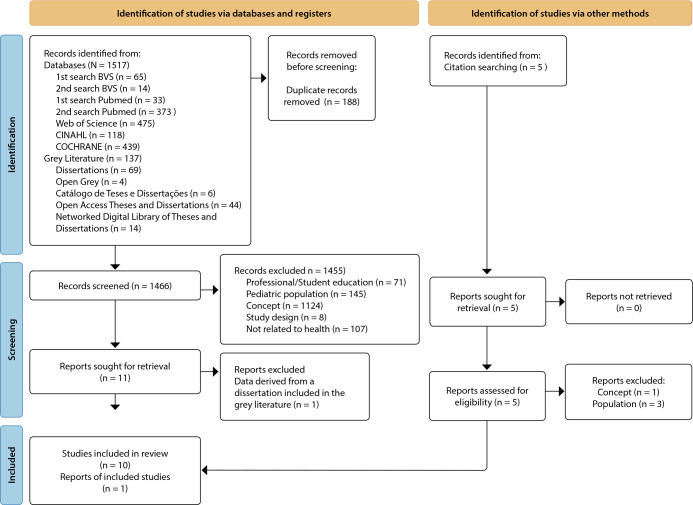
Flowchart for the Identification and Selection of Studies in Databases

After the identification and screening process of 1,517 studies, 11 studies were selected for analysis and inclusion, as presented in the flowchart based on PRISMA-ScR^(19)^ in Figure 1.

Chart 1 presents the characterization of the included studies, considering criteria such as the year of publication, country of origin, language, study design, theme addressed, main results, and risk of bias.

**Chart 1:** Characterization of the Included Studies, São Paulo, São Paulo, Brazil, 2023

Author, Year, Country, Language	Study Design	Theme	Main Results	Risk of Bias
Kamel, 2006, United Kingdom, English^(26)^	State of the Art	Technological Innovations for Health Education	1) The essence of podcasting is to create content that can be consumed whenever, wherever, and however the listener wishes. 2) Users can listen to podcasts on a computer or a portable device. 3) Podcasts can be created using speech software or real human voice. 4) Podcasts use Rich Site Summary (RSS). 5) Health-related podcasts are available for patients and the general public.	Low
Kamel, 2007, United Kingdom, English^(27)^	State of the Art	Technological Innovations for Health Education	1) Podcasts are digital files independent of time and location, accessible for free through podcast feed subscriptions or by automatic transfer to a computer or portable device for later listening or viewing. 2) Health podcasts are also used for communication with older, tech-savvy adults in the USA. 3) There are podcast search tools available. 4) Listener interaction creates a two-way communication experience, though instant interactivity is still somewhat illusory.	None
Turner-McGrievy, 2009, USA, English^(28)^	RCT	Obesity	1) Listening to podcasts led to healthy changes in 91% of listeners. 2) There was no difference in skin conductance level. 3) The website group found this tool less innovative compared to the podcast group. 4) The intervention group experienced greater weight loss and knowledge about weight loss, user control, and less cognitive load at the end of the intervention.	Moderate
Turner-McGrievy, 2011, USA, English^(29)^	RCT	Obesity	1) There was no difference in weight loss, self-reported food intake monitoring, or physical activity between groups at 6 months. 2) The intervention group downloaded more episodes than the control group. 3) The number of podcasts downloaded had a moderate relationship with weight loss in both groups.	Moderate
Ko, 2014, USA, English^(30)^	RCT	Obesity	1) Conceptual models were developed, and the study demonstrated the positive effect of a theory-based podcast on weight loss. 2) Information Control Theory and Cognitive Load Theory were related to elaboration, and elaboration was associated with weight loss. 3) Constructs of Social Cognitive Theory did not mediate weight loss.	Moderate
Hales, 2015, USA, English^(31)^	RCT	Obesity	1) The total points earned had a statistically significant relationship with the percentage of weight loss. 2) Awareness predicted a greater gain in points.	Moderate
Williams, 2015, USA, English^(32)^	Integrative Review	Alcohol Consumption	1) No studies on the topic were located. 2) Health podcasts are viable and cost-effective, but may be more time-consuming. 3) Humor and the use of narrative can be more entertaining to the listener.	None
Schroyen, 2017, France, English^(33)^	Cross-Sectional Study	Health Professional Guidance Elderly Population	1) Explanations for older patients had shorter statements and more repetitions, with fewer adverse effects mentioned, such as sex-related issues. 2) Professionals with a positive view of aging reduced the length of statements and the rate of words per minute in explanations to the older patient, but when the professional had a negative view of this process, these factors were observed in both explanations.	Moderate
Dahl, 2018, USA, English^(34)^	RCT	Weight Gain During Pregnancy	1) The difference in appropriate weight gain between the groups was not significant. 2) There was a significant difference in healthy eating, with higher healthy eating scores in the intervention group.	Moderate
Huberty, 2020, USA, English^(35)^	Single-Group Feasibility Study	Cancer	1) The average consumption was 103.2 minutes per week. 2) Nearly half of the participants reported enjoying the health education podcasts and were satisfied with the intervention. 3) There were no significant changes in cancer-related outcomes from the start to post-intervention.	Moderate
Silva, 2021, Brazil, Portuguese^(36)^	Methodological Development Study	Systemic Arterial Hypertension Elderly Population	Development of a nursing care podcast as a health education tool for the elderly, providing instructions on self-care, actions to improve quality of life, knowledge about the disease, and encouragement to adhere to both medicinal and non-medicinal therapies.	None

*RSS - Really Simple Syndication; USA - United States of America; RCT - Randomized Clinical Trial.*

Regarding the data sources, 54% of the selected studies are found in Pubmed (n=6)^(26-27,29-30,33,35)^, and 46% in grey literature databases - Open Thesis and Dissertations (n=4)^(28,31-32,34)^ and the Catalog of Theses and Dissertations (n=1)^(36)^.

**Figure 2:**
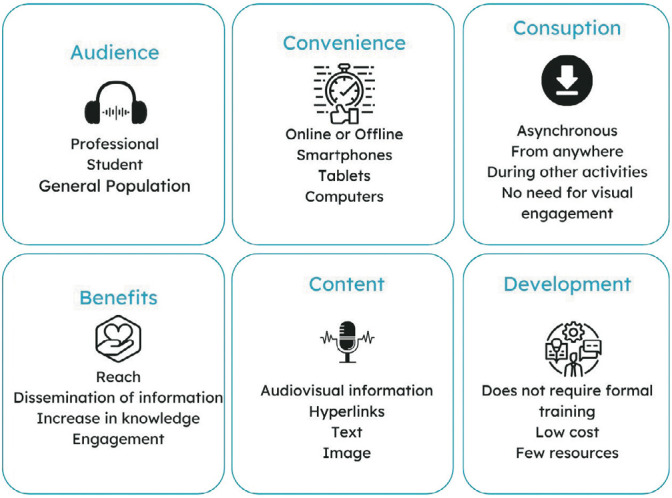
Characteristics of podcasts according to the included studies

Figure 2 summarizes the characteristics of the podcast related to audience, convenience, consumption, benefits, content, and development, abordados nos estudos incluídos nesta revisão^(27-28,34)^.

**Figure 3:**
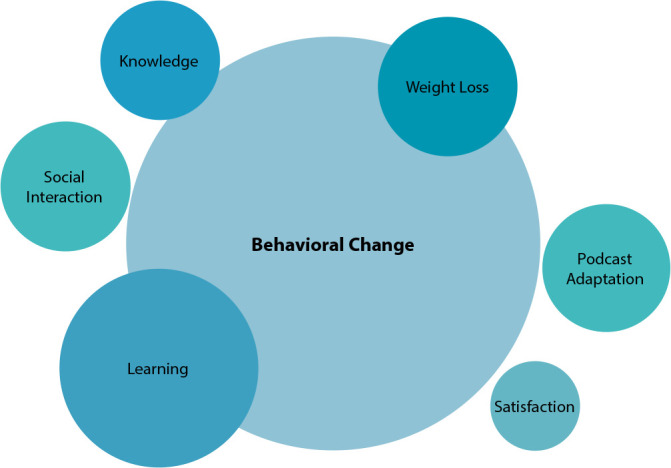
Outcomes related to podcast use

Figure 3 portrays the studied outcomes related to the use of podcasts, evaluated in the analyzed studies^(26-36)^.

## DISCUSSION

The qualities and advantages of podcasts have been discussed for over a decade^(27)^. With the increased use of the World Wide Web, there has also been a growth in the use of smartphones and mobile technologies, as well as in the access to and popularization of podcasts^(30)^. Upon analyzing the studies, it is evident that the growing use of eHealth strategies in the population requires the identification of effective communication channels and interventions^(34)^. Podcasts have been studied as one of these innovative strategies in increasing demand, showing positive characteristics for their use in health education^(26-36)^.

In this context, considering the increased consumption of this product, podcasts emerge as a communication tool available through digital media^(15,37-38)^, and due to the ease of their use^(28-33,36)^, they present themselves as a promising alternative to assist the educational process^(37)^. The authors emphasize the importance of podcasts as a crucial tool in disseminating information, significantly expanding the reach and accessibility of interventions^(34)^, given their ability to overcome geographical barriers^(27)^ and the convenience of the audio format^(28)^.

In turn, these characteristics grant podcasts a transformative role in promoting health knowledge and empowering individuals in managing their own health conditions^(15,18)^. However, although podcasts are a viable strategy for motivating users and complementing knowledge remotely^(39-40)^, Abreu highlights that in-person guidance is essential and can assist individuals in their self-care and treatment adherence. In this context, podcasts would serve as an additional educational material^(37)^, being precise and well-structured^(28)^. Supporting this assertion, an equivalence study involving 225 middle-aged women showed that groups involving consultations with nurses achieved better results in physical activities than the group that only received web-based guidance^(41)^. Therefore, it is important to reflect on the role of podcasts as an auxiliary tool in health education, as this difference (in-person versus remote) justifies the need for new studies with longer follow-up periods, comparing in-person guidance with podcast interventions.

Despite its importance, this review revealed that the research area of podcasts for health education is limited^(28,32,36)^, even though it is a strategy with various facilitating characteristics for its development and use^(27-36)^. Furthermore, studies have concluded that most podcasts developed are used as educational technology for students and professional development, with little evidence of their use to improve public health or as information technology for professionals in the health education process^(32,36)^.

Consequently, the lack of evidence also extends to the development of these resources, as studies suggest that most podcasts are created for health professionals or students in the field^(32,36)^, indicating the need for more research on the topic among health professionals^(36)^, especially because cost-effective and feasible educational interventions can reduce the impact of diseases worldwide^(42)^. The use of behavioral^(30)^ and communication^(32)^ theories in the development of podcasts has the potential to strengthen self-efficacy, behavioral capacity, and outcome expectations^(30)^, as well as to positively contribute to user satisfaction^(28)^. Moreover, this approach has been associated with more effective outcomes in increasing listener knowledge^(31)^.

In this context, detailed information on the methodology for creating health technologies is rare. A notable example is the study by Knudsen et al., which described the creation of a complex intervention for rheumatoid arthritis^(43)^. This study involved multiple stages in the development of e-learning resources, including podcasts, starting from a literature review and discussions with patient focus groups to transcription and content adaptation^(28,30,33-34)^. It is important to highlight that improved, well-structured, and theory-based podcasts can facilitate learning^(28,30)^.

Regarding the use of podcasts as a medium, they have proven effective for outcomes such as a sense of control over the learning pace, social interaction^(28)^, knowledge^(31)^, satisfaction^(28)^, motivation, behavioral change, and weight loss^(30-31)^. Additionally, they have been indicated as promoters of well-being, quality of life, autonomy, self-esteem, and a sense of belonging^(44)^. Therefore, podcasts should be considered as a strategy to increase treatment adherence and individual commitment, especially in more vulnerable populations^(29)^.

Concurrently, educating patients about behavioral change should include more than medication adherence, diet, and physical activity, as there are other factors still less disseminated through this medium that contribute to global public health challenges, such as smoking, a theme not addressed in the applied podcasts^(42)^. This review observed that podcasts primarily focused on topics like obesity, alcohol consumption, hypertension, cancer, and the elderly population. Therefore, it is inferred that new themes should be explored in patient education to ensure comprehensive guidance relevant to various populations.

In this scenario, despite the relevance of podcasts as a mass medium and the effectiveness of audio in delivering health information^(26-36)^, professionals and patients are not yet fully accustomed to this tool^(33)^. Therefore, professionals are encouraged to connect socially, as initiatives that transcend current communication barriers tend to impact patient health, as well as the way these professionals work^(27)^.

Regarding health-related podcasts for popular consumption, previous analyses of health content on web platforms have suggested the presence of erroneous information, with the accuracy of user-generated health podcasts being unknown^(32)^. However, it is crucial that the main stakeholders in media consumption, namely the patients^(31)^ and/or their families/caregivers, participate in its creation. This involvement demonstrates the partnership between professionals and patients in building this sociable health technology^(27)^, creating a quality educational product that resonates with the end user, potentially enhancing their engagement and satisfaction.

The studies found point out limitations in the samples^(28-29,33,35)^, highlighting small sizes and a lack of diversity in demographic aspects^(29,34-35)^, as well as their heterogeneous nature^(28,33)^. These characteristics prevent the generalization and, consequently, the reproducibility of the results. Due to this limitation, the question arose as to whether the characteristics of the sample could have influenced the use of the tool, the knowledge, and the understanding of the guidelines, raising the hypothesis that users of other ethnicities, incomes, and educational levels might achieve similar results^(28,33)^.

Additionally, some studies pointed out the superficiality in data analysis^(28)^, the absence of research on the topic^(36)^, and the selection of studies and synthesis of evidence with a certain degree of subjectivity^(32)^.

Finally, for future studies, research with longer intervention and follow-up periods^(28)^ was suggested, as well as the use of other tools and strategies in comparison to podcasts^(28,30)^, with the aim of verifying long-term results and the impact of each strategy^(30)^.

### Study limitations

This study presents several limitations that should be considered when interpreting its results. The heterogeneity of the included studies, in terms of both research designs and study populations, may have affected the ability to conduct robust comparative analyses.

Limitations related to the quality of the individual studies also warrant consideration, as a lack of standardization in measuring outcomes and the potential for selection bias may have impacted the validity of the results.

Finally, this study’s conclusions are based on a review of the existing literature up to the cut-off date. New developments or research published after this period may not have been incorporated.

### Contributions to Research and Health Fields

This comprehensive review study has added knowledge to the emerging field of using podcasts in health education, offering a synthesis of the evidence available to date. The research demonstrates the promising, innovative, and effective possibilities in the development and use of this medium as a strategy in the health education process, revealing that, although it is a valuable tool, it is not yet widely adopted or recognized.

Thus, the study serves as a valuable starting point for researchers interested in further exploring this innovative educational strategy.

## CONCLUSIONS

In terms of the characteristics of podcasts as an educational tool in health, it is clear that this medium transcends spatial and temporal limitations and emerges as an innovative, cost-effective, and easily implemented intervention. When structured effectively, a podcast can have a significant social impact, simplifying the educational process and disseminating health information.

Despite the scarcity of evidence, the use of podcasts as a tool has been effective for outcomes such as behavioral change, weight loss, satisfaction, and social interaction. Beyond these, podcasts have been recognized as resources promoting well-being, quality of life, autonomy, self-esteem, and belonging. Therefore, they should be considered as a strategy for increasing treatment adherence and individual commitment, particularly in more vulnerable populations.

Given the limited number of studies on the topic, many gaps still remain, particularly because the knowledge and description of podcast development methodologies are fields that have been scarcely explored. Furthermore, the analysis of the selected studies highlights that the themes addressed have been restricted, suggesting future studies should consider the relevance of this medium in generating satisfaction and enhancing the learning experiences of the main actors in the process: the professional and the patient.
